# Cytokine response in cerebrospinal fluid of meningitis patients and outcome associated with pneumococcal serotype

**DOI:** 10.1038/s41598-021-99190-3

**Published:** 2021-10-07

**Authors:** Annelies Müller, Diana B. Schramm, Jackie Kleynhans, Linda de Gouveia, Susan Meiring, Alban Ramette, Anne von Gottberg, Lucy Jane Hathaway

**Affiliations:** 1 Institute for Infectious Diseases, Faculty of Medicine, University of Bern, Bern, Switzerland; 2 Graduate School for Cellular and Biomedical Sciences, Faculty of Medicine, University of Bern, Bern, Switzerland; 3 National Institute for Communicable Diseases, Centre for HIV and STI’s, Johannesburg, South Africa; 4 School of Pathology, Faculty of Health Sciences, University of the Witwatersrand, Johannesburg, South Africa; 5 School of Public Health, Faculty of Health Sciences, University of the Witwatersrand, Johannesburg, South Africa; 6 Centre for Respiratory Diseases and Meningitis, National Institute for Communicable Diseases (NICD) of the National Health Laboratory Service (NHLS), Johannesburg, South Africa; 7 Division of Public Health Surveillance and Response, National Institute for Communicable Diseases (NICD) of the National Health Laboratory Service (NHLS), Johannesburg, South Africa

**Keywords:** Microbiology, Neuroscience, Diseases, Medical research

## Abstract

*Streptococcus pneumoniae* causes life-threatening meningitis. Its capsular polysaccharide determines the serotype and influences disease severity but the mechanism is largely unknown. Due to evidence of elevated cytokines levels in the meningeal inflammatory response, we measured 41 cytokines/chemokines and growth factors in cerebrospinal fluid (CSF) samples from 57 South African meningitis patients (collected in the period 2018–2019), with confirmed *S. pneumoniae* serotypes, using a multiplexed bead-based immunoassay. Based on multivariable Bayesian regression, using serotype 10A as a reference and after adjusting for HIV and age, we found IL-6 concentrations significantly lower in patients infected with serotypes 6D (undetectable) and 23A (1601 pg/ml), IL-8 concentrations significantly higher in those infected with 22A (40,459 pg/ml), 7F (32,400 pg/ml) and 15B/C (6845 pg/ml), and TNFα concentration significantly higher in those infected with serotype 18A (33,097 pg/ml). Although a relatively small number of clinical samples were available for this study and 28% of samples could not be assigned to a definitive serotype, our data suggests 15B/C worthy of monitoring during surveillance as it is associated with in-hospital case fatality and not included in the 13-valent polysaccharide conjugate vaccine, PCV13. Our data provides average CSF concentrations of a range of cytokines and growth factors for 18 different serotypes (14, 19F, 3, 6A, 7F, 19A, 8, 9N, 10A, 12F, 15B/C, 22F, 16F, 23A, 31, 18A, 6D, 22A) to serve as a basis for future studies investigating host–pathogen interaction during pneumococcal meningitis. We note that differences in induction of IL-8 between serotypes may be particularly worthy of future study.

## Introduction

*Streptococcus pneumoniae* is a major cause of pneumonia and invasive pneumococcal diseases (IPD) including life-threatening meningitis. An important virulence factor of the pneumococcus is its capsular polysaccharide (CPS). The CPS determines the serotype, is used for the manufacture of pneumococcal vaccines and plays a role in IPD^1^. Vaccines have reduced the burden of disease but protect only against a handful of the approximately 100 known circulating serotypes. As a result of vaccination, changes in the prevalence of the different serotypes has occurred, particularly an increase in those not included in the vaccines^2–4^. In addition, the odds ratio of causing invasive disease differs between serotypes^5,6^ and when IPD does occur, different serotypes have been observed to be responsible for different levels of disease severity^7–13^. A meta-analysis of disease severity by risk of death from IPD indicates that disease severity is a stable serotype-associated property, at least in the pre-PCV era^14^.

How the CPS affects disease severity is unclear. It has been suggested that the CPS can influence growth of the pneumococcus^15,16^ and that there are differences between serotypes in the ability to build a thick capsule. Both of these factors have been described both in vitro^15^ and in vivo^17^. Clearly though, the ability to cause severe disease is not only dependent on capsule size and bacterial growth but also on host–pathogen interactions, pre-existing immunity and the host immune response towards the different pneumococcal serotypes. Several studies suggest levels of cytokines such as interleukin (IL)-6, IL-8, IL-10, IL-1β and tumor necrosis factor (TNF)-α to be prognostic for the outcome of bacterial meningitis^18^ and certain pneumococcal serotypes have been associated with higher patient case-fatality ratios in large-scale epidemiological studies^19^. Cytokines and chemokines released during the host immune response function to not only recruit innate and adaptive immune cells but also promote inflammation and therefore play an important role in pneumococcal pathogenesis. Thus, it could be hypothesized that the invading pneumococcal serotype influences cytokine concentrations and profiles and that these could affect both disease severity and patient outcome (long-term sequelae and survival).

Interleukin-8 (IL-8) for example, has been noted by several studies to be an important player in the pathogenesis of pneumococcal diseases^18,20–23^ but in depth studies on the expression of IL-8 and other human inflammatory responses towards different pneumococcal serotypes and the resulting patient outcome, especially in meningitis cases, are scarce. Most are limited to animal models, to only a few markers and/or in the number of serotypes which are compared. This is likely due to the challenges of assessing the large number of known circulating serotypes within one study. Another reason may be the limited access to patient cerebrospinal fluid (CSF) samples to measure cytokines, to patient information and/or the difficulty of setting up experiments to analyze patient samples while considering all possible confounding factors. Despite these difficulties, an in depth understanding of the mechanisms by which pneumococcal serotypes cause differences in disease severity will be important to guide and improve future vaccine and treatment designs.

In this study we provide the average concentrations of 41 cytokines and growth factors produced in response to 18 different serotypes in patients with laboratory-confirmed pneumococcal meningitis. We aimed to determine the relationship between pneumococcal serotypes and (1) the inflammatory profile in residual CSF of South African patients with laboratory-confirmed pneumococcal meningitis and (2) the severity of disease (measured by in-hospital outcome) to establish whether serotypes associated with a high case-fatality ratio (CFR) in a time of routine PCV vaccination, also cause a distinct inflammatory reaction from the host which in turn affects the severity of disease. We highlight the difficulties, but also the advantages and insights, that our approach may bring, as a basis for the planning of future large-scale studies.

## Methods

### Study population

The study population included patients of all ages enrolled in the South African GERMS-SA enhanced surveillance study from January 2018 to August 2019, with laboratory-confirmed pneumococcal meningitis diagnosed from a lumbar puncture performed by the attending doctor. Samples were collected during the period where routine PCV13 vaccination of infants in a 2 plus 1 schedule was established. South Africa introduced the 7-valent PCV in 2009 and in 2011 PCV7 was replaced by PCV13.

The GERMS-SA programme (http://www.webdev.nicd.ac.za/index.php/germs-sa/) is an active laboratory-based surveillance programme for which information on bacterial and fungal pathogens, including *Streptococcus pneumoniae,* is collected. We chose 10 sentinel surveillance sites from provinces across South Africa (Eastern Cape, Free State, Gauteng, Kwazulu-Natal, Mpumalanga, North West and Western Cape) which had more than 10 cases of laboratory-confirmed pneumococcal meningitis cases with CSF available and cultured specimen in both 2014 and 2015. All sites chosen were part of the enhanced surveillance for which detailed patient information was available. Informed consent for analysis of CSF was collected from patients and/or guardians for both collection of data and analysis of residual CSF. Due to ethical reasons relating to the collection of CSF samples from healthy patients a control group was not included in this study. All research was performed in accordance with the relevant guidelines and regulations.

### Ethics

The GERMS-SA surveillance study was approved by the Human Research Ethics Committee (Medical), University of Witwatersrand (clearance numbers M140159, M081117, M021042) and additional permission for this study was granted by the Human Research Ethics Committee (Medical), University of Witwatersrand (clearance number M180101). Where required, approval was granted by provincial and university ethics committees (Clearance numbers: Free state 230,408–011 / 180/09A; Kwazulu-Natal BF130/11 and HRKM024/09; Western Cape REC REF 115/2009, N04/01/001). Permission for analysis of data in Switzerland was obtained from the Cantonal Ethics Commission Bern (Kantonale Ethikkommission für die Forschung, KEK, Bern, Project Number 2018–01,172).

### Sample collection and storage

Routine CSF sample collection and analysis is as follows. CSF patient samples were collected at the request of the attending clinician. After completion of diagnostic procedures any residual CSF was collected by the site surveillance officers if the presence of *S. pneumoniae* was confirmed by the local laboratory. Normal diagnostic procedures in all laboratories include microscopy (cell count and Gram stain), chemistry (protein, glucose and sodium levels), culture and antimicrobial susceptibility of any pathogen isolated. Where a raised white cell count and/or Gram-positive lanceolate diplococci were seen on Gram stain, a rapid antigen detection test is performed to confirm the presence of pneumococcal antigen. Pneumococcal isolates and/or residual CSF samples are then routinely submitted to the National Institute for Communicable Diseases (NICD) in Johannesburg, South Africa where full identification is carried out to confirm the species and serotyping of isolates by Quellung and polymerase chain reaction (PCR) is also performed. Real time-PCR (RT-PCR) for *lytA* gene detection is routinely performed on all samples, followed by a multiplex serotype PCR if positive^24^. The multiplex PCR detects and confirms 38 different pneumococcal serotypes and any samples that were not one of these 38 were called NEG38.

For this study, surveillance officers were given a standard operating procedure for the collection of CSF samples for this study. They were requested to store the residual CSF samples on site until further transport (at −20 °C or −80 °C depending on availability of equipment). The residual CSF samples were then transported from sentinel site laboratories of collection to the NICD. To avoid unnecessary freeze–thaw cycles all samples were transported on dry ice and immediately stored at −80 °C upon arrival at the NICD until further multiplex analysis. Of 860 pneumococcal meningitis patients at the selected sentinel sites during the study period, from whom CSF was collected for diagnostic purposes, 76 residual CSF samples were available for collection and analysis for this study. 12 samples had insufficient residual CSF (minimum 200 µl required) to perform multiplex analysis and were thus excluded from analysis. All samples were analyzed within 1 year of collection. After multiplex analysis, 5 samples were excluded due to time between collection and freezing of sample exceeding 48 h. Another 2 samples were excluded because upon follow-up it was confirmed that the infecting agent was not *S. pneumoniae*. Specimens which were NEG38, serotypes included in pool G (29, 34, 35, 42 and 47) and those which were not distinguishable (PCR positive but not distinguishable between individual serotypes) were grouped into "undefined non-vaccine serotypes". A total of n = 57 samples was included in the final analysis. Serotype 10A served as the reference serotype as it has previously been described as having an intermediate case fatality rate^13^ and has also previously been associated with increased risk of meningitis in different studies (summarized in^19^). In the present study, of the two patients with serotype 10A, one case was fatal and one survived. Serotypes of samples with missing outcome information included: 19F (n = 1), 3 (n = 1), 9 N (n = 1), 22F (n = 1), 6D (n = 1), 22A (n = 1), and undefined (n = 3). These samples were not included in the analysis of case fatality associated with serotype. We did not eliminate these samples from the analysis of inflammatory markers in an effort to keep the sample size and serotypes analyzed as large as possible.

### Analysis of inflammatory markers

We measured the concentrations of inflammatory markers in CSF using 39-plex and 5-plex microsphere-based multiplex assays (Human Custom ProcartaPlex 39-plex cat. number PPX-39-MX9HJM4 and 5-plex cat. number PPX-05-MXNKR67, Thermo Fisher Scientific, Waltham, MA, USA). The 39-plex immunoassay included the following markers: CD40-Ligand, Epithelial-derived neutrophil-activation protein (ENA)-78 (LIX), Eotaxin, Granulocyte-Colony stimulating factor (G-CSF)/CSF-3, Granulocyte–Macrophage(GM)-CSF, Growth related oncogene (Gro)-α/KC, Hepatocyte growth factor (HGF), Interferon (IFN)-α, IFN-γ, IL-10, IL-12p40, IL-13, IL-15, IL-17A, IL-1α, IL-1β, IL-2, IL-4, IL-5, IL-6, IL-7, IL-8, IFN-γ induced protein (IP)-10, IL-6 family cytokine (LIF), M-CSF, Monocyte chemotactic protein (MCP)-1, MCP-3, monokine induced by IFN-γ (MIG), Macrophage inflammatory protein (MIP)-1α, MIP-1β, Plasminogen activator inhibitor (PAI)-1, Platelet derived growth factor (PDGF)-BB, Resistin, Stem cell factor (SCF), TNF-α, TNF-related apoptosis-inducing ligand (TRAIL), Vascular endothelial growth factor (VEGF)-A, Neurotrophic growth factor (bNGF) and sFas-Ligand. The 5-plex immunoassay included the following markers: IFN-γ, IL-17F, IL-6, Transforming growth factor (TGF)-α, TNF-α. The markers were selected based on a previous publication which measured immune molecules in human CSF^25^ as well as from publications presenting data on their function and/or concentrations in inflammation, meningitis and/or CSF. The panels include a range of cytokines, chemokines and factors which play key roles in processes reflecting inflammatory pathways, during pneumococcal disease and/or which have been reported to be elevated in bacterial meningitis. Key functions of the chosen inflammatory markers with references to previous publications are summarized in Supplementary Table S1.

The assays were run according to the manufacturer’s instructions. Samples (25 µl) were measured undiluted and the standards and CSF samples run in duplicate. The 39-plex immunoassay was prioritized and if enough sample remained, the 5-plex immunoassay was additionally performed. This was possible for all except two samples which contained pneumococcal serotypes 6D and 18A. Beads were acquired on a Bio-Plex 200 instrument (Bio-Rad) and the fluorescence signal for 50 beads per analyte measured. The data was analyzed with Bio-Plex manager (version 6.1, Bio-Rad) capable of generating a five-parameter logistic (5-PL) curve fit. For statistical purposes, a value of zero was assigned to values with concentrations below the lower limit of quantification (LLOQ). To include concentrations with values above the upper limit of quantification for (ULOQ) statistical purposes, we assigned a new value where the concentration of the highest obtained value of the corresponding analyte was taken and a value of + 1000 added. Analyte concentrations are expressed as pg/ml.

### Statistical analysis

In-depth Bayesian univariate and multivariable modeling was performed specifically on IL-6, IL-8 and TNF-α, because both IL-6 and TNF-α are well described cytokines (see supplementary Table S1) and IL-8 because concentrations varied greatly between patients (data not shown). We chose Bayesian regression modeling to determine which serotypes may be associated with case fatality and varying cytokine concentrations. Due to the sample size limitation, a Bayesian framework may be more appropriate than conventional regression approaches^26^ to test for such associations. Due to the high prevalence of HIV amongst the South African patients and the known relationship between IPD incidence and HIV infection^27^, HIV was controlled for as a confounder. Age was a categorical variable with the following groups: below 5 years (10 samples), above or equal to 5 years (42 samples), unknown (5 samples).

Merging and cleaning of sample data and patient data from the GERMS-SA enhanced surveillance data was conducted using Stata 16.0 (StataCorp, College Station, Texas, USA). The risk of case fatality was evaluated as a function of serotype and associated co-variables via full Bayesian binomial model. Weakly informative priors were set by letting parameters follow Student's t distributions with 7 df, centered on 0 with a scale of 2.5 (default values). Concentrations of individual cytokines (IL-6, IL-8, TNF-α) were related to explanatory variables using Bayesian linear multivariable regression with drawing 4000 posterior samples. TNF-α values were log-transformed to conform to normality. In each model, a total of four Markov chains were used, each based on 1000 transitions using 10 leapfrog steps per transition, a warmup phase of 1000 and further sampling of 1000 steps. Bayesian regression analyses were conducted using the function stan_glm (Bayesian Generalized linear modeling) available in the R package *rstanarm*^28^. To ensure valid inferences, convergence to the target distributions of the Markov chains (the independent Markov chains were initialized with diffuse starting values for the parameters and were sampled until all values for Rhat were below 1.1) was checked. Effective Samples Size (ESS) for each estimated parameter were all above 1000, which is largely above what is recommended for applied regression analyses^28^.

## Results

A total of 57 samples were included in the final analysis. Amongst those with known gender information, 46% (24/52) were female. For those with available HIV status, 79% (34/43) were positive. Of those with available age information the age range was 0 to 69 years and the three most represented age groups were patients aged 25–44 years (25/52, 44%), 45–64 years (10/52, 18%) and those aged < 1 year (8/52, 14%). The three most common serotypes were serotype 8 (7/57, 12%), serotype 3 (5/57, 9%) and serotype 12F (5/57, 9%). Of the 18 serotypes identified, five were PCV serotypes: serotypes 3, 6A, 7F, 14 and 19A, but of these only serotype 3 was represented in more than 5% of the samples. Isolates/specimens which were non-typable, included in the pool G serotypes, or undistinguishable, together made up 28% (16/57) of the cases. Of the cases with available outcome information (17/48, 35%) had a fatal outcome (Supplementary Table S2). Detailed Bayesian statistics was performed on three cytokines with key roles in inflammation in bacterial meningitis: IL-6, IL-8 and TNFα. We did not perform detailed Bayesian statistics on all inflammatory markers to avoid increasing the risk of family-wise error due to multiple testing. However, concentrations per serotype of all inflammatory markers can be found in Supplementary Table S3. Overall, the analysis of cytokine concentrations indicated that CSF from meningitis patients with serotypes 6D and 23A had low levels of IL-6 whereas those with serotypes 22A, 7F and 15B/C had high levels of IL-8 and with serotype 18A had high concentrations of TNFα.

### Univariate and multivariable analysis of IL-6 concentration between different serotypes

**Figure 1:**
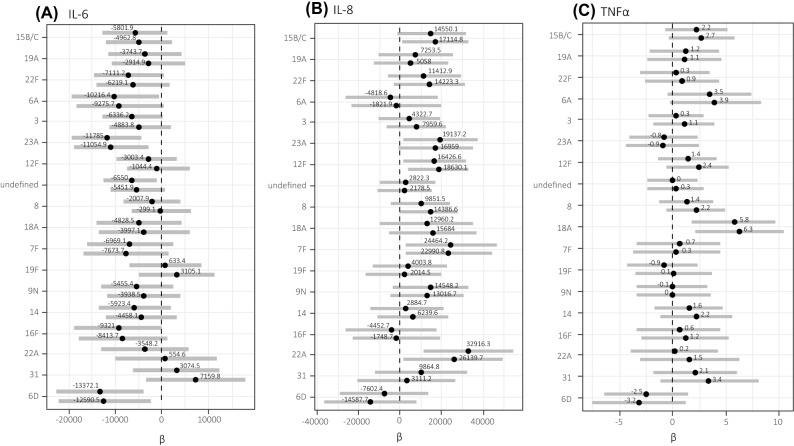
Serotypes associated with varying concentrations of (**A**) IL-6, (**B**) IL-8 and (**C**) TNF-α in cerebrospinal fluid (South African meningitis patients, 2018–2019). The 90% highest density intervals (HDI) of the posterior distributions of the estimated regression coefficients (β) are depicted as horizontal bars and dots indicate their medians, for both unadjusted (upper bars) and adjusted (lower bars; covariables HIV status and patient age) full Bayesian multivariable linear regression models. Serotypes are sorted from top to bottom in decreasing risk of case fatality. Serotype 10A was chosen as ad-hoc reference, for which β was set to 0. TNF-α concentrations were log-transformed to conform to normality prior to analysis. n = 57. This figure was generated using R (R Core Team (2019)). R: A language and environment for statistical computing. R Foundation for Statistical Computing, Vienna, Austria. https://www.R-project.org/).

On multivariable analysis controlling for age and HIV status and compared to serotype 10A, serotype 6D (coefficient [coef]: −12,712.82, highest density interval lower to upper bound [HDI]: −22,977.67, −2514.49) and 23A (coef: -10,979.91, HDI: −19,201.69, −2869.32) were associated with lower levels of IL-6 in the CSF of meningitis patients (Fig. 1A, Supplementary Table S4, Bayesian R^2^ = 0.44). On univariate analysis and compared to meningitis patients infected with serotype 10A, those infected with 6D, 23A, 6A, 16F and 3 were associated with lower levels of IL-6 concentrations in the CSF (Fig. 1A, Supplementary Table S4). The group of "undefined serotypes" including those non-typable, not distinguishable by PCR and those in pool G were significantly associated with a lower IL-6 concentration on univariate analysis but not on multivariable analysis.

### Univariate and multivariable analysis of IL-8 concentration between different serotypes

On multivariable analysis controlling for age and HIV status and compared to serotype 10A, serotypes 22A (coef: 26,090.77, HDI: 2389.31, 48,894.44), 7F (coef: 23,255.71, HDI: 1374.87, 44,902.99) and 15B/C (coef: 17,050.5, HDI: 614.6, 33,345.73) were associated with higher levels of IL-8 in the CSF of meningitis patients (Fig. 1B, Bayesian R^2^ = 0.44, Supplementary Table S5). Serotypes significantly associated with higher levels of IL-8 in CSF compared to 10A on univariate analysis were serotypes 22A, 7F, 23A and 12F (Fig. 1B, Bayesian R^2^ = 0.44, Supplementary Table S5). Although serotype 6D had noticeably lower levels of IL-8 (Supplementary Table S3), this was not significant on either univariate or multivariable analysis (Fig. 1B, Supplementary Table S5).

### Univariate and multivariable analysis of TNFα concentration between different serotypes

Compared to serotype 10A, on multivariable analysis controlling for age and HIV status, we found serotype 18A to be associated with higher concentration of TNFα concentration (coef: 6.22, HDI: 2.08, 10.31) in the CSF of meningitis patients (Fig. 1C, Bayesian R^2^ = 0.40, Supplementary Table S6). We observed a lower TNFα concentration in the meningitis patient infected with serotype 6D (Supplementary Table S3) which was, however, not significant upon univariate and multivariable analysis (Fig. 1C, Supplementary Table S6). We did not see any pattern between serotypes associated with case fatality and cytokine concentrations.

### Univariate and multivariable analysis of case fatality measured by in-hospital outcome between different serotypes

**Figure 2:**
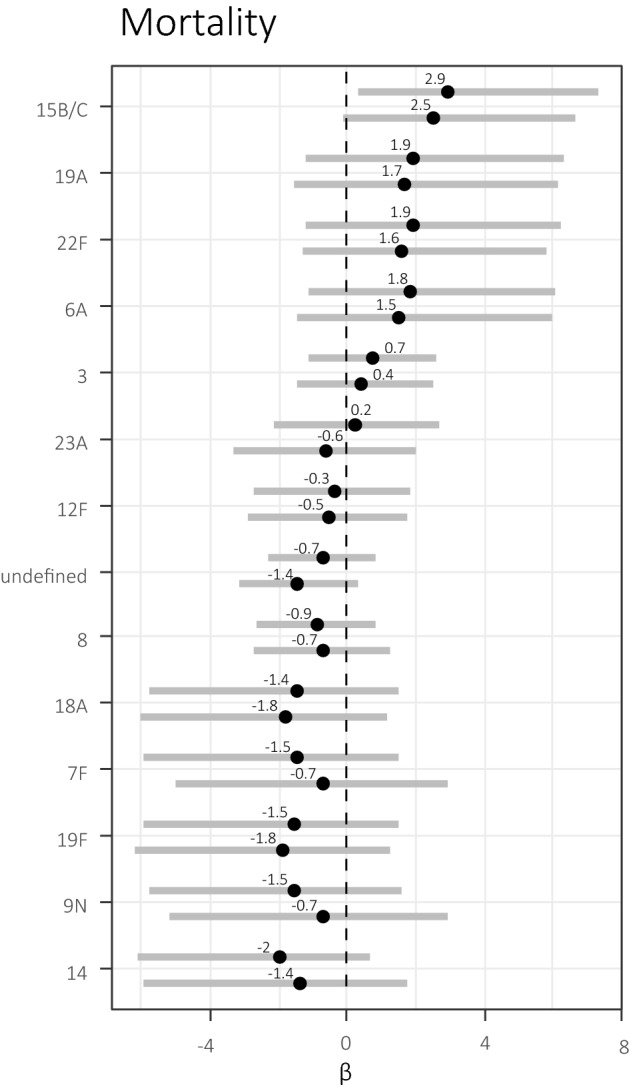
Serotypes associated with higher case fatality risk (South African meningitis patients, 2018–2019). The 90% highest density intervals (HDI) of the posterior distributions of the estimated regression coefficients (β) are depicted as horizontal bars and dots indicate their medians, for both unadjusted (upper bars) and adjusted (lower bars; covariables HIV status and patient age) full Bayesian multinomial logit models. Serotypes are sorted from top to bottom in decreasing risk of case fatality as compared to the case fatality rate of the ad-hoc reference serotype 10A, for which β was set to 0. n = 57. This figure was generated using R (R Core Team (2019)). R: A language and environment for statistical computing. R Foundation for Statistical Computing, Vienna, Austria. https://www.R-project.org/).

Using Bayesian statistics, compared to serotype 10A (1 out of 2 fatal) on a univariate but not multivariable analysis of serotypes associated with case fatality (in-hospital outcome), we found serotype 15B/C (3 of 3 fatal) to be significantly associated with case fatality (coeff 2.94, HDI: 0.34–7.35) (Fig. 2 Bayesian R^2^ = 0.28, Supplementary Table S7). No other serotypes were significantly associated with case fatality compared to serotype 10A. Apart from serotype 15B/C, serotypes which showed a trend towards being associated with case fatality on both uni- and multivariable analysis were serotypes 19A, 22F, 6A and 3. Serotypes 8, 7F, 19F, 9 N and 14 showed a trend towards being less associated with case fatality compared to 10A.

## Discussion

We hypothesized that serotypes associated with severe disease would cause a strong inflammatory reaction, and therefore high concentrations of inflammatory cytokines especially IL-6, IL-8 and TNFα compared to serotypes associated with less severe disease. To test this, we looked for an association of serotype with case fatality. Due to the small sample size (n = 57), we opted for Bayesian regression analyses instead of classical frequentist approaches and found serotype 15B/C to be significantly associated with case fatality in our cohort. In descending order, we found serotypes 15B/C, 19A, 22F, 6A and 3 to show a (non-significant) trend towards being associated with case fatality compared to 10A. This is similar to previous publications which also found these serotypes associated with either severe disease and/or a higher CFR^19^. We also see serotype 15B/C being associated with case fatality in a large epidemiological study including all IPD cases in all ages in the vaccine era (2012–2018) which we have conducted (manuscript in preparation). These findings further support the association between 15B/C and case fatality in this study. Based on this conclusion, and in combination with knowledge from previous publications we hypothesized that especially serotypes 15B/C, 19A, 6A and 3 would cause a strong inflammatory reaction with high concentrations of IL-6, IL-8 and TNFα.

We were able to determine differences in the average concentration of 41 different inflammatory markers in the CSF of pneumococcal meningitis patients infected with different serotypes (14, 19F, 3, 6A, 7F, 19A, 8, 9N, 10A, 12F, 15B/C, 22F, 16F, 23A, 31, 18A, 6D, 22A) in a period of established PCV13 vaccination. We found that compared to meningitis patients infected with serotype 10A, the CSF concentration of IL-6 was significantly lower in those infected with 23A and 6D, the IL-8 concentration was significantly higher in those infected with 22A, 7F and 15B/C; and the TNFα concentration was significantly higher in those infected with serotype 18A. Concentrations of other inflammatory markers (especially ENA-78, G-CSF/CSF-3, Gro-α/KC, HGF, IFN-γ, IL-10, IL-1β, IP-10, LIF, M-CSF, MCP-1, MIG, MIP-1α, MIP-1β, PAI-1, Resistin, and VEGF-A) varied greatly between patients. A distinct inflammatory profile of serotypes associated with severe disease remains to be determined in a larger study.

In line with our hypothesis, we found serotype 15B/C to be significantly associated with a higher IL-8 concentration in CSF compared to serotype 10A. We did not find concentrations of IL-6, IL-8 or TNFα in CSF of meningitis patients infected with other serotypes including those previously associated with case fatality and/or severe disease, to be significantly different to those infected with serotype 10A. IL-6 is an acute phase response cytokine^29^. It is possible that the CSF samples were taken after the peak of the IL-6 response and differences between serotypes missed. Contrary to our expectation, we also found serotype 7F to be associated with relatively high levels of IL-8. Serotype 7F is described as highly invasive rather than a common colonizer and in a previous in vivo experiment caused less severe disease than serotype 6B^17^. IL-8 is an important chemoattractant for neutrophils in humans and has been shown to play a role in the pathogenesis of pneumococcal disease^18,20–22,30,31^. Two in vitro studies describe pneumolysin as a factor influencing the IL-8 concentration^20,32^ while two other studies (in vitro and in vivo) describe NanA (a sialidase expressed by pneumococci) to contribute to changes in IL-8 concentrations^21,33^. Both these mechanisms may also contribute to the varying amounts of IL-8 which we see between patient samples. That the serotype influences the host IL-8 concentration was also observed in another study^34^. The latter study was limited to three serotypes (1, 3 and 9V) and our results confirm and extend these results with additional serotypes. Overall, although a larger study would be required to confirm the effect of serotype on the secretion of IL-8 (as well as IL-6 and TNFα) and on patient outcome, we did find that IL-8 differed significantly between more than two serotypes. Whether changes in IL-8 concentrations are related to serotype alone, a specific genetic trait of the patient or a combination of different factors (including HIV therapy as mentioned in^35^) or other bacteria factors, such as haemolytic activity of pneumolysin or other virulence factors as described above, remains to be determined.

We faced two very important challenges in this study, both of which show the complexity and difficulties of setting up a study such as this. The first and in our opinion the most important, was that we were only able to collect a small number of samples per serotype. This means (1) we cannot rule out the effect of the host genetic background as well as other confounding factors on the reaction towards pneumococcal serotypes, (2) that we may have a selection bias and are missing serotypes for which samples were not collected and (3) that we cannot yet make final conclusions about differences of cytokine concentrations caused by different serotypes. However, a strength of this study is that, instead of using frequentist statistics which are often based on maximum likelihood estimation and often offer insufficient power with small sample sizes, we used a Bayesian statistical framework with weak priors, to better quantify the amount of information in the data^26,36^. A second important challenge was that we were not able to control the time point of CSF collection or how the samples were processed. CSF samples are difficult to obtain, both ethically and logistically. A big advantage of this study was that we obtained residual CSF from lumbar punctures routinely performed for diagnosis from patients already enrolled in the GERMS-SA surveillance programme. This allowed us to perform analysis on samples for which clinical data was available and meant there was no additional suffering for the patient due to CSF collection. The disadvantage of this is that the concentrations measured indicate the inflammatory marker concentrations upon patient admission and this may be a different time point in disease for each patient. In any future studies it will be necessary to rule out the effect of the host genetic background by increasing the sample size to include hundreds of CSF samples as well as addressing other mentioned challenges above. Future studies would also benefit from the collection of blood samples at the same time as CSF. Implementing all of these suggestions will be challenging for a single study and therefore may be addressed by multiple smaller studies analyzing each one of these aspects.

In conclusion, we were able to determine the concentrations of 41 inflammatory markers in the CSF of 57 South African meningitis patients infected with 18 different pneumococcal serotypes in an era of established PCV vaccination. We showed that even with a limited sample size, Bayesian statistics is appropriate to gain valuable insights and obtain information useful to guide future studies. We aimed to contribute to the pathophysiological understanding of pneumococcal meningitis, specifically the relationship between serotype, cytokine levels in the CSF and the outcome. We hope that this data will be useful for planning future larger studies, for example for power analysis to estimate sample sizes required. The panel of cytokine data should prove useful comparison in future studies of pneumococcal meningitis and other inflammatory diseases of the CSF.

We note that serotype 15B/C may be associated with in-hospital case fatality. 15B/C is not a component of the current PCV13 vaccine and so we propose that it is of interest in surveillance and future vaccine design. We did not find a clear correlation between serotype, in-hospital case fatality and the CSF concentration of IL-6, IL-8 and TNFα in CSF of meningitis patients but note that IL-8, which was at a relatively high concentration in patients infected with serotype 15B/C, may be of particular interest for future studies.

## Supplementary Information

Supplementary Information.

## Data Availability

All data generated or analysed during this study are included in this published article and its supplementary information files.

## Abbreviations

CFR: Case-fatality rate
CPS: Capsular polysaccharide
CSF: Cerebrospinal fluid
IL: Interleukin
IPD: Invasive pneumococcal disease
LLOQ: Lower limit of quantification
PCV: Polysaccharide conjugate vaccine
TNF: Tumor necrosis factor
ULOQ: Upper limit of quantification
