# Supplementary material for: Cytokine response in cerebrospinal fluid of meningitis patients and outcome associated with pneumococcal serotype
Source: Sci Rep. 2021 Oct 7;11:19920. doi: 10.1038/s41598-021-99190-3 (PMC8497479; doi:10.1038/s41598-021-99190-3)
Supplement: Supplementary file 1 — Supplementary Information. [file 41598_2021_99190_MOESM1_ESM.pdf]

S1 Table1: Grouping and background information on inflammatory markers chosen for analysis in cerebrospinal fluid of South African pneumococcal meningitis patients

Inflammatory marker*	Group**	Other names	Known functions	Reference(s)
NGF-β	2		Neurotrophic growth factor which has positive effects on axon growth, cell survival and postnatal development	[1]
CD40-Ligand	4		Member of the TNF receptor family and essential for antibody response to thymus dependent antigens,<br>Important for the generation of a robust T-cell dependent memory response,	[2, 3]
ENA-78 (LIX)	3	CXCL5	Proposed to play a role in recruitment of neutrophils, promotion of angiogenesis and remodeling of connective tissue,<br>In mice, indication that CXCL5 contributes to resistance of pneumococcal infection,	[4]
Eotaxin	3	CCL11	Potent and eosinophilic specific chemoattractant,<br>Proposed to play a role in heightened inflammatory response of aged mice when exposed to *S, pneumoniae*,	[5]
G-CSF	2	CSF-3	Involved in the stimulation of proliferation and differentiation of hematopoietic progenitor cells,<br>In a mouse model, proposed to improve spatial learning and to stimulate neurogenesis after pneumococcal meningitis,	[6]
GM-CSF	1	CSF-2	High levels at sites of inflammation and autoimmunity,<br>Cytolytic activity of a pneumococcal D39 strain was found to induce increased expression of genes encoding GM-CSF,	[7-9]
Gro-α	3	KC<br>CXCL1<br>MGSA-α	In a sequencing study to determine the role of genetic variation on pneumococcal meningitis susceptibility and outcome, a Gro-α gene had a strong signal for susceptibility,<br>Suggested to be a major chemoattractant of leukocytes into the subarachnoid space during meningitis,	[10, 11]
HGF	2	-	Shown to regulate acute and chronic inflammation in different disease models, and found to be elevated in CSF of patients with acute bacteria/probable bacterial meningitis compared to nonbacterial CNS infections,	[12-14]
IFN-α	1	-	Mainly involved in innate immunity and secreted by immune and non-immune cells in answer to viral infections,	[15]
IFN-γ	1	-	Primarily secreted by activated T-cells and natural killer cells, Can promote macrophage activation, mediate antiviral and antibacterial immunity, enhance antigen presentation, orchestrate activation of innate immune system, coordinate lymphocyte-endothelium interaction, regulate Th1/Th2 balance and control cellular proliferation and apoptosis,<br>Observations indicate that there is an association between IFN-γ driven acute brain pathology and the long-term neurological sequelae resulting from pneumococcal meningitis,	[16, 17]
IL-10	1	-	Cytokine with anti-inflammatory properties and may play an important role in preventing damage to the host by limiting the immune response towards pathogens,<br>Proposed to modulate the magnitude of the neutrophil influx,<br>Higher concentrations in severe sepsis patients compared to sepsis patients has been shown,	[5, 18, 19]
IL-12p40	1	-	The p40 subunit is distantly related to the IL-6 receptor,<br>Upregulation via binding of IFN-γ (produced upon TLR activation),	[20]
IL-13	1	-	Involved in allergic inflammation, mainly involved in parasitic infections,	[21, 22]
IL-15	1	-	Pleiotropic cytokine which has functions including regulating tissue repair, modulating inflammation and activating natural killer cells, upon infections with bacteria, IL-15 expression increases,	[23, 24]
IL-17A	1	CTLA-8	Plays a protective role in host defense against certain pathogens at the epithelial and mucosal barriers, A deficiency in IL-17A/F has been related to a high susceptibility to *Streptococcus pneumoniae* and other infections in humans, A dysregulation of IL-17A/F production can result in pro-inflammatory cytokine expression and chronic inflammation, IL-17 has been shown to be elevated in CSF of children with bacterial meningitis and is proposed to play a key role in neutrophil infiltration into the CSF,	[25-27]
IL-17F	1	-	A deficiency in IL-17A/F has been related to a high susceptibility to *Streptococcus pneumoniae* and other infections in humans, A	[26]

Inflammatory marker*	Group**	Other names	Known functions	Reference(s)
			dysregulation of IL-17A/F production can result in pro-inflammatory cytokine expression and chronic inflammation,	
IL-1α	1	-	IL-1α is a dual function cytokine which binds both to DNA and to its cell membrane receptor, In the nucleus, it serves to increase gene expression of IL-8 for example,<br>Has been shown to play a role in BBB disruption,	[28-30]
IL-1β	1	-	Induced by microbial products via the TLR ligands, shown to be increased within a short time after exposure of immune cells to LPS: Higher concentrations in severe sepsis compared to sepsis patients has been shown, It has been described as a marker of inflammatory activity in the CSF and is related to clinical outcome parameters in bacterial meningitis,<br>In a murine model of pneumococcal meningitis, the upregulation in was independent of CSF leukocytosis, pneumococcal pneumolysin and H_2O_2 but was induced by pneumococcal cell wall fragments,	[18, 28, 31-34]
IL-2	1	-	Plays a role in regulation of immune activation and homeostasis, HIV infection has been shown to lead to a deficiency in IL-2 production, IN a comparison study of cytokines in CSF between pneumococcal and meningococcal meningitis, IL-2 concentrations were higher in pneumococcal meningitis,	[35, 36]
IL-4	1	-	Proposed to play a role during late stages of pneumococcal meningitis, Additionally, to playing a role in immunity, evidence points to a role in memory and learning in the normal brain,	[37, 38]
IL-5	1	-	Plays a role in hematopoiesis and largely plays a role in allergic or eosinophilic inflammatory diseases,	[9]
IL-6	1	-	Pro-inflammatory cytokine, has been shown useful as a factor for diagnostic, clinical severity and prognosis, Higher concentrations in severe sepsis compared to sepsis patients, and in patients with fatal outcome has been shown as well as a strong negative relationship of conc, with neurological sequelae of concentration in plasma,<br>Is a well-studied cytokine in animal models of bacterial meningitis,	[18, 30, 36, 39-42]
IL-7	1	-	Important for T-cell development and survival as well as homeostasis of mature T-cells, proposed to play an essential role in the anti-polysaccharide response towards *streptococcus pneumoniae*,	[43-45]
IL-8	3	CXCL8	Important chemoattractant for neutrophils, Higher concentrations in severe sepsis compared to sepsis patients has been shown,<br>IL-8 differences in concentration between serotypes in a rabbit model of pneumococcal meningitis, Pneumolysin has been found to be a major factor influencing the IL-8 response from human nasopharyngeal epithelial,	[11, 18, 46-48]
IP-10	3	CXCL10	Reported to be enhanced in patients co-infected with *Streptococcus pneumoniae* and Influenza and suggested to be a biomarker capable of severity prediction various diseases,	[38, 49, 50]
LIF	1	-	Member of the interleukin-6 cytokine family which all activate the signal transducer and activator of transcription 3 (STAT3), STAT3 influences stem and progenitor cells,	[51]
MCP-1	3	CCL2<br>MCAF<br>TDCF	Plays a role in BBB disruption through recruitment of monocytes/macrophages, MCP-1 plasma levels have been suggested to be associated with sepsis induced organ dysfunction,	[52-54]
MCP-3	3	CCL7	MCP-3 is a chemokine which attracts Macrophages which may play a role in chronic inflammation,	[55, 56]
M-CSF	2	CSF-1	Known to regulate tissue macrophage homeostasis, Evidence is emerging that, in mice, the M-CSF signaling could directly control the development and maintenance of microglia cells,	[57]
MIG	3	CXCL9	Is involved in the regulation of leukocyte trafficking and is induced by IFN-γ, It has been shown to induce antibacterial activity *in vitro,*	[58]
MIP-1α	3	CCL3<br>LD78α	Has been suggested to be beneficial in *Streptococcus pneumoniae* induced meningitis, Increase of MIP-1α was demonstrated in meningitis *in vivo* and in glial cells *in vitro* in reaction to *Streptococcus pneumoniae,*	[59, 60]
MIP-1β	3	CCL4	Has both inflammatory and chemokinetic properties and binds to the CCR5 receptor, it is one of the major HIV suppressive factors produced by CD8+ T-cells, It is a major chemoattractant for natural killer cells,	[60-62]
PAI-1	4	Serpin	PAI-1 is a serine protease inhibitor (modulation of serine protease activities is essential for coagulation), Serpins are present in both host and pathogen, Host serpins may directly inhibit pathogen proteases and pathogen binding, One study mentioned a positive correlation between PAI-1 concentration in CSF and the BBB breakdown,	[63-65]

Inflammatory marker*	Group**	Other names	Known functions	Reference(s)
PDGF-BB	2	-	PDGF's play an important role in angiogenesis and have been introduced in clinics as a wound healing therapy,	[66]
Resistin	4	ADSF	Resistin was originally known as an adipocyte specific hormone and is thought to be a link between obesity, insulin resistance and diabetes, there has been a report of astrocytes expressing resistin, High levels tend to be associated with a pro-inflammatory effect and have been shown to stimulate pro-inflammatory cytokines,	[67, 68]
SCF	4	-	SCF is important for the maturation and infiltration of mast cells,	[69]
sFas-Ligand	4	-	The Fas-Ligand is a member of the TNF-superfamily, the primary function is the induction of apoptosis, It is a well-known marker of apoptosis, In patients with bacteremia, it has been associated with high sequential organ failure assessment scores,	[70] [48]
TGF-α	2	-	TGF-α from astrocytes has been reported to play a neuroprotective role against neurotoxic insults and has been shown to be upregulated during brain injury,	[71-75]
TNF-α	1	-	Up-regulation in neurons and astrocytes in the hippocampus during pneumococcal meningitis in murine models has been noted and is a marker of inflammatory activity in the CSF, It has also been noted as a parameter correlating to the clinical outcome in bacterial meningitis,	[18, 34, 76-78]
TRAIL	4	-	A member of the TNF-superfamily, it is expressed by various immune cells and is a well-known marker of apoptosis, in an experimental model of *Streptococcus pneumoniae* caused meningitis, TRAIL-/- mice showed prolonged inflammation, augmented clinical impairment and increased hippocampal apoptosis	[79, 80]
VEGF-A	2	-	Stimulates angiogenesis, vascular repair and regeneration and may also promote neuronal functions such as neurogenesis, neuronal migration neuronal survival and axon guidance,	[81]

***Abbreviations:** Epithelial-derived neutrophil-activation protein (ENA), Granulocyte (G), Colony stimulating factor (CSF), Macrophage (M), Growth related oncogene (Gro), Hepatocyte growth factor (HGF), Interferon (IFN), Interleukin (IL), Interferon (IFN), induced protein (IP), IL-6 family cytokine (LIF), Monocyte chemotactic protein (MCP), Monokine induced by IFN-γ (MIG), Macrophage inflammatory protein (MIP), Plasminogen activator inhibitor (PAI), Platelet derived growth factor (PDGF), Stem cell factor (SCF), Tumor necrosis factor (TNF), TNF-related apoptosis-inducing ligand (TRAIL), Vascular endothelial growth factor (VEGF), Neurotrophic growth factor (NGF), Transforming growth factor (TGF)
****Group 1:** cytokines, Group 2: growth factors, Group 3: chemokines, Group 4: other inflammatory markers [82]

REFERENCES

1. Sofroniew, M.V., C.L. Howe, and W.C. Mobley, *Nerve growth factor signaling, neuroprotection, and neural repair.* Annu Rev Neurosci, 2001. **24**: p. 1217-81.
2. Elgueta, R., et al., *Molecular mechanism and function of CD40/CD40L engagement in the immune system.* Immunol Rev, 2009. **229**(1): p. 152-72.
3. Jha, V. and E.N. Janoff, *Complementary Role of CD4+ T Cells in Response to Pneumococcal Polysaccharide Vaccines in Humans.* Vaccines (Basel), 2019. **7**(1).
4. Mancuso, R.I., et al., *Impaired expression of CXCL5 and matrix metalloproteinases in the lungs of mice with high susceptibility to Streptococcus pneumoniae infection.* Immun Inflamm Dis, 2018. **6**(1): p. 128-142.
5. Williams, A.E., et al., *Enhanced inflammation in aged mice following infection with Streptococcus pneumoniae is associated with decreased IL-10 and augmented chemokine production.* Am J Physiol Lung Cell Mol Physiol, 2015. **308**(6): p. L539-49.
6. Schmidt, A.K., et al., *Adjuvant granulocyte colony-stimulating factor therapy results in improved spatial learning and stimulates hippocampal neurogenesis in a mouse model of pneumococcal meningitis.* J Neuropathol Exp Neurol, 2015. **74**(1): p. 85-94.
7. Hamilton, J.A., *Colony-stimulating factors in inflammation and autoimmunity.* Nat Rev Immunol, 2008. **8**(7): p. 533-44.
8. Harvey, R.M., et al., *The impact of pneumolysin on the macrophage response to Streptococcus pneumoniae is strain-dependent.* PLoS One, 2014. **9**(8): p. e103625.
9. Dougan, M., G. Dranoff, and S.K. Dougan, *GM-CSF, IL-3, and IL-5 Family of Cytokines: Regulators of Inflammation.* Immunity, 2019. **50**(4): p. 796-811.
10. Ferwerda, B., et al., *Variation of 46 Innate Immune Genes Evaluated for their Contribution in Pneumococcal Meningitis Susceptibility and Outcome.* EBioMedicine, 2016. **10**: p. 77-84.
11. Sprenger, H., et al., *Chemokines in the cerebrospinal fluid of patients with meningitis.* Clin Immunol Immunopathol, 1996. **80**(2): p. 155-61.
12. Molnarfi, N., et al., *Hepatocyte growth factor: A regulator of inflammation and autoimmunity.* Autoimmun Rev, 2015. **14**(4): p. 293-303.
13. Nayeri, F., et al., *Hepatocyte growth factor levels in cerebrospinal fluid: a comparison between acute bacterial and nonbacterial meningitis.* J Infect Dis, 2000. **181**(6): p. 2092-4.
14. Kern, M.A., et al., *Concentrations of hepatocyte growth factor in cerebrospinal fluid under normal and different pathological conditions.* Cytokine, 2001. **14**(3): p. 170-6.
15. Gougeon, M.L. and J.P. Herbeuval, *IFN-alpha and TRAIL: a double edge sword in HIV-1 disease?* Exp Cell Res, 2012. **318**(11): p. 1260-8.
16. Too, L.K., et al., *The pro-inflammatory cytokine interferon-gamma is an important driver of neuropathology and behavioural sequelae in experimental pneumococcal meningitis.* Brain Behav Immun, 2014. **40**: p. 252-68.
17. Tau, G. and P. Rothman, *Biologic functions of the IFN-gamma receptors.* Allergy, 1999. **54**(12): p. 1233-51.
18. Perdomo-Celis, F., et al., *Patterns of Local and Systemic Cytokines in Bacterial Meningitis and its Relation with Severity and Long-Term Sequelae.* Biomark Insights, 2015. **10**: p. 125-31.
19. Saraiva, M. and A. O'Garra, *The regulation of IL-10 production by immune cells.* Nat Rev Immunol, 2010. **10**(3): p. 170-81.
20. Zundler, S. and M.F. Neurath, *Interleukin-12: Functional activities and implications for disease.* Cytokine Growth Factor Rev, 2015. **26**(5): p. 559-68.
21. Junttila, I.S., *Tuning the Cytokine Responses: An Update on Interleukin (IL)-4 and IL-13 Receptor Complexes.* Front Immunol, 2018. **9**: p. 888.
22. Wynn, T.A., *IL-13 effector functions.* Annu Rev Immunol, 2003. **21**: p. 425-56.
23. Perera, P.Y., et al., *The role of interleukin-15 in inflammation and immune responses to infection: implications for its therapeutic use.* Microbes Infect, 2012. **14**(3): p. 247-61.
24. Patidar, M., N. Yadav, and S.K. Dalai, *Interleukin 15: A key cytokine for immunotherapy.* Cytokine Growth Factor Rev, 2016. **31**: p. 49-59.

25. McGeachy, M.J., D.J. Cua, and S.L. Gaffen, *The IL-17 Family of Cytokines in Health and Disease.* Immunity, 2019. **50**(4): p. 892-906.
26. Jin, W. and C. Dong, *IL-17 cytokines in immunity and inflammation.* Emerg Microbes Infect, 2013. **2**(9): p. e60.
27. Asano, T., et al., *IL-17 is elevated in cerebrospinal fluids in bacterial meningitis in children.* Cytokine, 2010. **51**(1): p. 101-6.
28. Akdis, M., et al., *Interleukins (from IL-1 to IL-38), interferons, transforming growth factor beta, and TNF-alpha: Receptors, functions, and roles in diseases.* J Allergy Clin Immunol, 2016. **138**(4): p. 984-1010.
29. Di Paolo, N.C. and D.M. Shayakhmetov, *Interleukin 1alpha and the inflammatory process.* Nat Immunol, 2016. **17**(8): p. 906-13.
30. Al-Obaidi, M.M.J. and M.N.M. Desa, *Mechanisms of Blood Brain Barrier Disruption by Different Types of Bacteria, and Bacterial-Host Interactions Facilitate the Bacterial Pathogen Invading the Brain.* Cell Mol Neurobiol, 2018. **38**(7): p. 1349-1368.
31. Leiva, L.E., et al., *Up-regulation of CD40 ligand and induction of a Th2 response in children immunized with pneumococcal polysaccharide vaccines.* Clin Diagn Lab Immunol, 2001. **8**(2): p. 233-40.
32. Dinarello, C.A., *Overview of the IL-1 family in innate inflammation and acquired immunity.* Immunol Rev, 2018. **281**(1): p. 8-27.
33. McKelvie, B., et al., *Fatal pneumococcal meningitis in a 7-year-old girl with interleukin-1 receptor activated kinase deficiency (IRAK-4) despite prophylactic antibiotic and IgG responses to Streptococcus pneumoniae vaccines.* J Clin Immunol, 2014. **34**(3): p. 267-71.
34. Izadpanah, K., et al., *Brain parenchymal TNF-alpha and IL-1beta induction in experimental pneumococcal meningitis.* J Neuroimmunol, 2014. **276**(1-2): p. 104-11.
35. Gaffen, S.L. and K.D. Liu, *Overview of interleukin-2 function, production and clinical applications.* Cytokine, 2004. **28**(3): p. 109-23.
36. Coutinho, L.G., et al., *Cerebrospinal-fluid cytokine and chemokine profile in patients with pneumococcal and meningococcal meningitis.* BMC Infect Dis, 2013. **13**: p. 326.
37. Gadani, S.P., et al., *IL-4 in the brain: a cytokine to remember.* J Immunol, 2012. **189**(9): p. 4213-9.
38. Klein, M., et al., *Protein expression pattern in experimental pneumococcal meningitis.* Microbes Infect, 2006. **8**(4): p. 974-83.
39. Takahashi, W., et al., *Usefulness of interleukin 6 levels in the cerebrospinal fluid for the diagnosis of bacterial meningitis.* J Crit Care, 2014. **29**(4): p. 693 e1-6.
40. Tanaka, T., M. Narazaki, and T. Kishimoto, *IL-6 in inflammation, immunity, and disease.* Cold Spring Harb Perspect Biol, 2014. **6**(10): p. a016295.
41. Schaaf, B., et al., *The interleukin-6 -174 promoter polymorphism is associated with extrapulmonary bacterial dissemination in Streptococcus pneumoniae infection.* Cytokine, 2005. **31**(4): p. 324-8.
42. Grandgirard, D., et al., *The causative pathogen determines the inflammatory profile in cerebrospinal fluid and outcome in patients with bacterial meningitis.* Mediators Inflamm, 2013. **2013**: p. 312476.
43. ElKassar, N. and R.E. Gress, *An overview of IL-7 biology and its use in immunotherapy.* J Immunotoxicol, 2010. **7**(1): p. 1-7.
44. Hassane, M., et al., *Interleukin-7 protects against bacterial respiratory infection by promoting IL-17A-producing innate T-cell response.* Mucosal Immunol, 2020. **13**(1): p. 128-139.
45. Shriner, A.K., et al., *IL-7-dependent B lymphocytes are essential for the anti-polysaccharide response and protective immunity to Streptococcus pneumoniae.* J Immunol, 2010. **185**(1): p. 525-31.
46. Baumgartner, D., et al., *Clinical Streptococcus pneumoniae isolates induce differing CXCL8 responses from human nasopharyngeal epithelial cells which are reduced by liposomes.* BMC Microbiol, 2016. **16**(1): p. 154.

47. Ostergaard, C., et al., *Influence of the blood bacterial load on the meningeal inflammatory response in Streptococcus pneumoniae meningitis.* BMC Infect Dis, 2006. **6**: p. 78.
48. Ostergaard, C., et al., *Differences in survival, brain damage, and cerebrospinal fluid cytokine kinetics due to meningitis caused by 3 different Streptococcus pneumoniae serotypes: evaluation in humans and in 2 experimental models.* J Infect Dis, 2004. **190**(7): p. 1212-20.
49. Liu, M., et al., *CXCL10/IP-10 in infectious diseases pathogenesis and potential therapeutic implications.* Cytokine Growth Factor Rev, 2011. **22**(3): p. 121-30.
50. Hoffmann, J., et al., *Viral and bacterial co-infection in severe pneumonia triggers innate immune responses and specifically enhances IP-10: a translational study.* Sci Rep, 2016. **6**: p. 38532.
51. Onishi, K. and P.W. Zandstra, *LIF signaling in stem cells and development.* Development, 2015. **142**(13): p. 2230-6.
52. Deshmane, S.L., et al., *Monocyte chemoattractant protein-1 (MCP-1): an overview.* J Interferon Cytokine Res, 2009. **29**(6): p. 313-26.
53. Holub, M., et al., *Selected Biomarkers Correlate with the Origin and Severity of Sepsis.* Mediators Inflamm, 2018. **2018**: p. 7028267.
54. Bossink, A.W., et al., *Plasma levels of the chemokines monocyte chemotactic proteins-1 and -2 are elevated in human sepsis.* Blood, 1995. **86**(10): p. 3841-7.
55. Ben-Baruch, A., et al., *Monocyte chemotactic protein-3 (MCP3) interacts with multiple leukocyte receptors. C-C CKR1, a receptor for macrophage inflammatory protein-1 alpha/Rantes, is also a functional receptor for MCP3.* J Biol Chem, 1995. **270**(38): p. 22123-8.
56. Menten, P., A. Wuyts, and J. Van Damme, *Monocyte chemotactic protein-3.* Eur Cytokine Netw, 2001. **12**(4): p. 554-60.
57. Chitu, V., et al., *Emerging Roles for CSF-1 Receptor and its Ligands in the Nervous System.* Trends Neurosci, 2016. **39**(6): p. 378-393.
58. Eliasson, M., et al., *Streptococcus pneumoniae induces expression of the antibacterial CXC chemokine MIG/CXCL9 via MyD88-dependent signaling in a murine model of airway infection.* Microbes Infect, 2010. **12**(7): p. 565-73.
59. Aust, V., et al., *Lack of chemokine (C-C motif) ligand 3 leads to decreased survival and reduced immune response after bacterial meningitis.* Cytokine, 2018. **111**: p. 246-254.
60. Menten, P., A. Wuyts, and J. Van Damme, *Macrophage inflammatory protein-1.* Cytokine Growth Factor Rev, 2002. **13**(6): p. 455-81.
61. Maghazachi, A.A., *Role of chemokines in the biology of natural killer cells.* Curr Top Microbiol Immunol, 2010. **341**: p. 37-58.
62. Maurer, M. and E. von Stebut, *Macrophage inflammatory protein-1.* Int J Biochem Cell Biol, 2004. **36**(10): p. 1882-6.
63. Winkler, F., et al., *Role of the urokinase plasminogen activator system in patients with bacterial meningitis.* Neurology, 2002. **59**(9): p. 1350-5.
64. Bao, J., et al., *Serpin functions in host-pathogen interactions.* PeerJ, 2018. **6**: p. e4557.
65. Horrevoets, A.J., *Plasminogen activator inhibitor 1 (PAI-1): in vitro activities and clinical relevance.* Br J Haematol, 2004. **125**(1): p. 12-23.
66. Andrae, J., R. Gallini, and C. Betsholtz, *Role of platelet-derived growth factors in physiology and medicine.* Genes Dev, 2008. **22**(10): p. 1276-312.
67. Acquarone, E., et al., *Resistin: A reappraisal.* Mech Ageing Dev, 2019. **178**: p. 46-63.
68. Morash, B.A., et al., *Resistin expression and regulation in mouse pituitary.* FEBS Lett, 2002. **526**(1-3): p. 26-30.
69. Theoharides, T.C., et al., *Mast cells and inflammation.* Biochim Biophys Acta, 2012. **1822**(1): p. 21-33.
70. Huttunen, R., et al., *Apoptosis markers soluble Fas (sFas), Fas Ligand (FasL) and sFas/FasL ratio in patients with bacteremia: a prospective cohort study.* J Infect, 2012. **64**(3): p. 276-81.
71. Karki, P., et al., *Transcriptional Regulation of Human Transforming Growth Factor-alpha in Astrocytes.* Mol Neurobiol, 2017. **54**(2): p. 964-976.

72. Yuen, E.C. and W.C. Mobley, *Therapeutic potential of neurotrophic factors for neurological disorders.* Ann Neurol, 1996. **40**(3): p. 346-54.
73. White, R.E., et al., *Transforming growth factor alpha transforms astrocytes to a growth-supportive phenotype after spinal cord injury.* J Neurosci, 2011. **31**(42): p. 15173-87.
74. Ma, Y.J., et al., *Region-specific regulation of transforming growth factor alpha (TGF alpha) gene expression in astrocytes of the neuroendocrine brain.* J Neurosci, 1994. **14**(9): p. 5644-51.
75. Junier, M.P., *What role(s) for TGFalpha in the central nervous system?* Prog Neurobiol, 2000. **62**(5): p. 443-73.
76. Kastenbauer, S., et al., *Patterns of protein expression in infectious meningitis: a cerebrospinal fluid protein array analysis.* J Neuroimmunol, 2005. **164**(1-2): p. 134-9.
77. Pfeffer, K., *Biological functions of tumor necrosis factor cytokines and their receptors.* Cytokine Growth Factor Rev, 2003. **14**(3-4): p. 185-91.
78. Barichello, T., et al., *Tumor necrosis factor alpha (TNF-alpha) levels in the brain and cerebrospinal fluid after meningitis induced by Streptococcus pneumoniae.* Neurosci Lett, 2009. **467**(3): p. 217-9.
79. Falschlehner, C., U. Schaefer, and H. Walczak, *Following TRAIL's path in the immune system.* Immunology, 2009. **127**(2): p. 145-54.
80. Hoffmann, O., et al., *TRAIL limits excessive host immune responses in bacterial meningitis.* J Clin Invest, 2007. **117**(7): p. 2004-13.
81. Mackenzie, F. and C. Ruhrberg, *Diverse roles for VEGF-A in the nervous system.* Development, 2012. **139**(8): p. 1371-80.
82. *Cellular and Molecular Immunology*. 2011, New York, NY: [Saunders].

S2 Table: Demographic and clinical characteristics of patients with meningitis included in the analysis

Variable		n/N	%
PROVINCE	Eastern Cape	11/57	19.3
	Free State	8/57	14.0
	Gauteng	13/57	22.8
	KwaZulu-Natal	3/57	5.3
	Mpumalanga	9/57	15.8
	North West	2/57	3.5
	Western Cape	7/57	12.3
	Missing	4/57	7.0
GENDER	Female	24/57	42.1
	Male	28/57	49.1
	Missing	5/57	8.8
HIV	Negative	9/57	15.8
	Positive	34/57	59.7
	Missing	14/57	24.6
AGE CATEGORY	<1y	8/57	14.0
	1-4y	2/57	3.5
	5-14y	4/57	7.0
	15-24y	1/57	1.8
	25-44y	25/57	43.9
	45-64y	10/57	17.5
	>=65y	2/57	3.5
	Missing	5/57	8.8
RACE	Other	10/57	17.5
	Black	36/57	63.2
	Missing	11/57	19.3
SEROTYPE	14	2/57	3.5
	19F	2/57	3.5
	3	5/57	8.8
	6A	1/57	1.8
	7F	1/57	1.8
	19A	2/57	3.5
	8	7/57	12.3
	9N	2/57	3.5
	10A	2/57	3.5
	12F	5/57	8.8
	15B/C	3/57	5.3
	22F	2/57	3.5
	16F	1/57	1.8
	23A	2/57	3.5
	31	1/57	1.8
	18A	1/57	1.8
	6D	1/57	1.8
	22A	1/57	1.8
	Undefined*	16/57	28.1
IN-HOSPITAL OUTCOME	Recovered	31/57	54.4

Variable		n/N	%
	Died	17/57	29.8
	Missing	9/57	15.8
UNDERLYING MEDICAL CONDITION**	No	32/57	56.1
	Yes	11/57	19.3
	Missing	14/57	24.6

*Undefined serotypes include pool G serotypes, non-typables and serotypes not distinguishable by polymerase chain reaction
**Underlying medical condition includes pre-disposing conditions defined as any one or more of the following: burns, chronic lung disease (including asthma, chronic obstructive pulmonary disorder, cystic fibrosis), chronic liver disease, chronic renal disease, cardiac conditions (including valvular disease and heart failure), cerebrovascular accident, stroke, neuromuscular diseases, cerebral palsy, metabolic diseases (including diabetes mellitus), head injury, surgery, cerebrospinal fluid leaks, ventricular shunts, cochlear implants, primary immunodeficiency conditions, complement deficiency, immunosuppression treatment (steroids/chemo/cancer treatment) protein-energy malnutrition, functional or anatomic asplenia (including sickle cell disease), malignancy, organ transplant, chromosomal conditions (including down syndrome), prematurity and aplastic anaemia,

S3 Table: Cytokine concentration (pg/ml) of residual cerebrospinal fluid collected from South African meningitis patients (2018 – 2019) according to diagnosed serotype

	Serotype*																		
**Marker****	**14**	**19F**	**3**	**6A**	**7F**	**19A**	**8**	**9N**	**10A**	**12F**	**15B/C**	**22F**	**16F**	**23A**	**31**	**22A**	**18A**	**6D**	**Undefined*****
	**n = 2**	**n = 2**	**n = 5**	**n = 1**	**n = 1**	**n = 2**	**n = 7**	**n = 2**	**n = 2**	**n = 5**	**n = 3**	**n = 2**	**n = 1**	**n = 2**	**n = 1**	**n = 1**	**n = 1**	**n = 1**	**n=16**
CD40	17.51	62.30	11.72	16.45	1.01	15.21	26.74	14.07	22.22	16.61	25.83	18.22	14.55	2.95	70.64	29.65	13.53	1.01	15.90
ENA	89.46	180.33	43.53	34.37	0.00	194.13	1777.73	111.62	513.09	2875.83	4482.06	1134.80	38.21	321.93	228.54	14890.95	12.50	0.00	1068.85
Eotaxin	7.59	6.97	5.52	12.62	3.68	6.45	5.96	4.03	6.28	4.67	7.43	5.14	3.80	2.15	22.19	8.59	4.21	2.52	4.51
G-CSF/CSF-3	909.24	307.49	302.52	46.98	0.00	220.67	2335.61	1936.42	7308.39	1967.74	1353.57	309.90	0.00	0.00	1576.50	14057.75	63.61	0.00	1323.91
GM-CSF	81.30	124.59	95.09	242.21	0.00	16.86	53.52	31.33	135.53	45.42	0.00	0.00	0.00	0.00	719.56	0.00	0.00	0.00	8.65
Gro-α/KC	1213.69	207.17	122.94	618.29	13.11	673.53	626.84	35.91	704.02	611.79	610.24	816.47	320.54	283.93	1146.57	2153.01	51.81	0.00	355.11
HGF	7419.35	4950.27	7449.37	9138.10	25494.03	17889.07	11119.48	9868.78	5954.55	9812.87	200030.70	20399.02	7630.99	7137.01	1676.77	282928.90	15617.22	38.60	7300.61
IFNα	1.85	2.36	1.62	1.97	2.47	1.65	2.08	1.36	2.72	1.42	1.39	1.73	0.76	0.22	9.63	1.43	3.30	0.00	0.92
IFNγ	256.25	37.62	758.25	443.71	6079.63	5718.53	66.06	127.89	140.26	209.97	3258.66	246.15	23.57	7.82	151.28	304.63	696.84	0.00	302.49
IL-10	637.30	159.34	947.48	2745.65	56.16	914.08	1718.05	2959.07	673.73	2971.62	868.56	3127.14	1699.70	72.04	383.49	2065.47	5614.52	0.00	617.06
IL-12p40	128.21	8.03	45.33	43.07	59.31	15.28	70.69	22.02	36.36	137.33	36.05	38.25	28.29	19.64	11.09	7.29	123.82	1.28	38.76
IL-13	3.19	1.85	5.28	7.42	1.30	6.07	5.35	1.65	6.01	3.45	1.67	4.97	0.00	0.89	19.74	2.96	0.00	7.53	2.34
IL-15	26.19	39.63	23.86	18.78	28.42	21.92	35.24	13.92	44.93	16.39	23.70	24.25	15.46	11.71	118.23	40.01	9.14	16.16	18.53
IL-17A	46.34	109.06	438.26	30.06	32.96	277.02	109.73	24.97	120.22	33.95	11.69	13.80	15.99	2.87	158.69	499.52	29.81	0.46	73.05
IL-1α	63.18	21.51	30.50	34.16	64.88	38.58	81.44	25.27	42.62	93.08	57.02	36.56	18.35	31.36	39.09	38.14	197.82	0.24	87.42
IL-1β	2410.06	185.55	1385.04	1479.06	522.10	1281.66	3628.90	2624.89	2239.10	4239.05	895.56	741.61	415.00	372.62	540.10	731.82	4459.04	0.07	1933.67
IL-2	69.45	48.03	34.68	58.02	2.91	25.48	29.13	21.57	53.10	28.92	13.27	6.91	9.40	1.81	203.88	17.24	20.39	0.00	17.31
IL-4	43.19	38.12	27.54	28.51	12.74	36.06	38.89	22.57	58.68	32.75	24.36	18.02	19.67	11.22	144.84	42.26	15.26	0.00	19.19
IL-5	25.10	12.41	0.00	65.43	24.85	16.81	28.72	12.30	3.94	9.53	4.10	0.00	0.00	40.32	12.30	0.00	13.43	0.00	7.68
IL-6	7523.75	14133.38	7887.79	3247.48	6455.15	10615.81	8957.10	10498.69	13779.67	9342.78	7600.77	6337.28	4019.86	1601.81	16592.72	9996.30	8535.20	0.00	6820.39
IL-7	6.33	12.74	9.23	7.99	0.10	10.39	5.73	1.49	12.20	2.51	3.14	1.18	0.72	0.00	70.32	3.93	0.57	0.42	2.89
IL-8	10452.04	11543.47	18060.53	2971.11	32400.29	27471.77	15753.68	23461.58	6845.97	24200.88	22175.01	19028.41	2893.99	26910.03	17387.48	40459.70	20289.43	0.00	10465.79
IP-10	1433.52	1141.39	292.63	113.55	471.30	3176.56	1895.22	126.81	2153.84	1120.59	1457.60	1597.39	49.20	1526.00	2377.08	3641.95	125.47	0.00	1098.30
LIF	972.63	803.89	1309.27	680.85	1855.50	1963.51	1577.95	1983.48	1265.19	1671.34	2051.83	1339.86	638.36	1682.57	1165.22	2139.84	2514.00	0.00	1267.19
M-CSF	507.08	57.42	1453.00	717.94	1053.10	1274.42	991.29	1349.47	273.80	905.74	1588.13	1472.53	531.76	343.13	210.29	410.57	1865.73	0.00	503.59
MCP-1	97696.90	2977.03	7438.13	4377.07	193532.80	193532.80	88489.87	26759.60	4520.24	72099.27	8956.57	97619.28	8701.55	99204.34	7587.86	193532.80	8357.30	18.73	27198.27
MCP-3	120.71	221.00	64.28	84.60	250.20	184.27	265.54	62.78	359.68	66.21	382.34	302.56	82.62	17.48	37.20	564.52	62.65	41.61	155.53
MIG	1270.56	785.93	892.28	1540.30	4886.99	2610.45	863.34	1108.90	710.87	1270.46	3165.75	2510.67	226.64	2054.78	280.58	3000.25	3073.44	0.00	979.06
MIP1α	1390.05	386.65	470.50	636.37	37805.91	1424.68	1225.09	799.70	754.55	703.40	709.40	2070.82	302.85	19403.85	1687.24	746.36	1243.44	0.00	597.41
MIP-1β	4511627.00	3069.22	3008634.00	5462.89	9024200.00	88879.97	882864.30	15255.90	4284.27	488985.10	15239.24	711033.10	6063.62	9024200.00	12227.99	69539.58	9024200.00	0.00	593139.70
PAI-1	15062.43	18875.71	19278.55	16235.39	29995.02	32633.63	23295.22	27402.62	28659.42	18813.65	93134.44	40163.86	17755.00	4439.60	14506.07	241860.00	25421.54	0.00	16527.27
PDGF-BB	19.89	94.93	73.85	40.95	0.00	91.63	36.60	0.00	99.85	8.17	75.56	0.00	0.00	0.00	560.58	76.58	0.00	56.45	17.01
Resistin	21975.63	29249.79	21728.63	4204.02	20857.36	43816.86	30576.92	43816.86	43816.86	27086.53	25989.33	14795.84	4757.23	11614.90	9725.65	16330.26	10895.39	0.00	17210.88
SCF	3.86	11.31	2.06	13.65	1.22	3.14	16.12	1.97	8.75	3.15	1.96	2.33	1.09	0.00	10.08	21.22	1.68	0.49	6.35
TNF-α	3757.15	53.03	707.99	3473.62	200.88	1953.84	1402.38	288.35	98.76	1093.40	1301.85	221.06	196.58	67.88	882.82	133.28	33097.01	8.65	653.24
TRAIL	35.55	34.80	40.99	35.90	100.52	48.86	39.16	23.97	45.43	44.89	86.44	71.91	15.26	74.03	35.30	153.81	104.58	0.00	28.04
VEGF-A	2702.28	1314.72	822.80	4521.62	8988.32	8961.75	1929.13	843.39	329.18	2977.90	7460.57	1526.67	396.48	14898.85	667.67	408.27	1535.36	0.15	2368.60
bNGF	13.91	8.61	9.04	40.03	31.80	24.97	20.19	13.41	10.21	19.54	66.07	60.50	19.12	3.45	22.23	48.55	34.29	50.51	10.70
sFas Ligand	26.99	1.29	6.23	17.03	38.70	52.13	57.52	11.90	20.87	18.55	23.03	26.53	2.98	0.00	45.44	129.33	14.73	0.00	11.46
IL-17F	0.42	0.76	6.12	0.00	21.23	46.66	1.56	5.76	0.52	2.26	1.15	0.00	0.77	0.80	0.00	32.64	N/A	N/A	1.11
TGF-α	379.87	49.49	239.96	379.38	129.37	440.84	312.01	865.09	144.24	392.46	251.20	181.15	341.43	427.19	77.60	426.83	N/A	N/A	247.21

*** Serotype:** listed are the values in pg/ml measured in the CSF of patients for which serotyping was possible. If more than one patient had the same serotype then the average of concentration is listed. Of a total of 57 samples, 16 samples where not possible to serotype or were not possible to distinguish,

****Marker analysis:** We measured the concentrations of inflammatory markers in CSF using a magnetic bead-based 39-plex and 5-plex immunoassay (Human Custom ProcartaPlex 39-plex cat. number PPX-39-MX9HJM4 and 5-plex cat. number PPX-05-MXNKR67, Thermo Fisher Scientific, Waltham, MA, USA). The 39-plex immunoassay included the following markers: CD40-Ligand, Epithelial-derived neutrophil-activation protein (ENA)-78 (LIX), Eotaxin, Granulocyte-Colony stimulating factor (G-CSF)/CSF-3, Granulocyte-Macrophage(GM)-CSF, Growth related oncogene (Gro)-α/KC, Hepatocyte growth factor (HGF), Interferon (IFN)-α, IFN-γ, IL-10, IL-12p40, IL-13, IL-15, IL-17A, IL-1α, IL-1β, IL-2, IL-4, IL-5, IL-6, IL-7, IL-8, IFN-γ induced protein (IP)-10, IL-6 family cytokine (LIF), M-CSF, Monocyte chemotactic protein (MCP)-1, MCP-3, monokine induced by IFN-γ (MIG), Macrophage inflammatory protein (MIP)-1α, MIP-1β, Plasminogen activator inhibitor (PAI)-1, Platelet derived growth factor (PDGF)-BB, Resistin, Stem cell factor (SCF), TNF-α, TNF-related apoptosis-inducing ligand (TRAIL), Vascular endothelial growth factor (VEGF)-A, Neurotrophic growth factor (bNGF) and sFas-Ligand. The 5-plex immunoassay included the following markers: IFN-γ, IL-17F, IL-6, Transforming growth factor (TGF)-α, TNF-α. The markers were chosen based on a previous publication which measured immune molecules in human CSF (Hornig, Gottschalk et al. 2016) and publications with data on their function and/or concentrations in inflammation, meningitis and/or CSF.

*****undefined:** includes pool G serotypes (29, 34, 35, 42 and 47), non-typables and serotypes not distinguishable by polymerase chain reaction; for two of the samples, it was not possible to determine IL17F and TGF-α values due to limited amount of available CSF, thus for these two markers the sample number is n = 14.

N/A: IL-17F and TGF-α determination was not possible due to limited amount of sample volume.

S4 Table Associations of serotypes with IL-6 concentrations

	Univariate			Adjusted*		
Serotype	**coefficient**	**HDI-L****	**HDI-H****	**coefficient**	**HDI-L****	**HDI-H****
10A	*Reference*	*Reference*	*Reference*	*Reference*	*Reference*	*Reference*
12F	-3311.72	-9540.95	3120.97	-1035.2	-8014.23	5717.68
14	-6283.22	-13419.21	1377.97	-4275.64	-12404.71	3594.25
15B/C	-6083.59	-12864.02	1012.27	-4930.73	-12371.63	2364.44
16F	-9745.65	-18727.47	-165.06	-8504.7	-18417.47	988.88
18A	-5291.19	-14535.82	3903	-4092.33	-13777.23	5267.04
19A	-4060.78	-11256.57	3569.2	-2804.94	-11013.53	4888.83
19F	397.12	-7051.84	8127.94	2978.48	-5291.28	11189.58
22A	-3659.03	-12848.41	6062.43	218.57	-10668.68	11353.3
22F	-7315.9	-14782.88	466.1	-6303.8	-14123.68	1644.33
23A	-12080.67	-19297.77	-4390.36	-10979.91	-19201.69	-2869.32
3	-6662.45	-12825.1	-120.9	-4791.38	-11942.64	1873.42
31	2739.84	-6518.99	11878.5	6866.76	-4106.6	17774.4
6A	-10482.07	-19853.7	-883.65	-9096.28	-18786.04	632.44
6D	-13650.48	-22880.48	-4407.92	-12712.82	-22977.67	-2514.49
7F	-7116.51	-16354.66	2000.97	-7460.98	-17258.2	1992.42
8	-2248.77	-8118.93	3897.07	-168.09	-6724.55	6152.8
9N	-5683.89	-13244.85	2131.55	-3756.32	-11898.71	4453.8
Undefined***	-6989.64	-12318.61	-1058.77	-5348.64	-11594.53	568.58

*Adjusted coefficient: adjusted for human immunodeficiency virus (HIV) status and age group
**HDI: Highest Density Interval of posterior distributions with "-L" = lower bound and "-H" = upper bound
***Undefined serotypes include pool G serotypes (29, 34, 35, 42 and 47), non-typables and serotypes not distinguishable by polymerase chain reaction

S5 Table Associations of serotypes with IL-8 concentrations

	Univariate			Adjusted*		
Serotype	**coefficient**	**HDI-L****	**HDI-H****	**coefficient**	**HDI-L****	**HDI-H****
10A	*Reference*	*Reference*	*Reference*	*Reference*	*Reference*	*Reference*
12F	16558.09	1939.89	30699.32	18361.19	2769.29	33223.73
14	2939.32	-14716.4	20849.48	6256.32	-11855	24113.37
15B/C	14656.28	-1334.57	30098.83	17050.5	614.6	33345.73
16F	-4810.28	-25911.5	16297.2	-2016.15	-23645.5	19078.65
18A	12641.69	-8343.97	33918.62	15097.41	-6887.35	37211.63
19A	7625.42	-10613.2	24321.47	5022.63	-13269.9	22839.28
19F	3928.54	-13214.9	21050.33	1768.36	-16920.5	20464.41
22A	32926.77	11196.61	53776.67	26090.77	2389.31	48894.44
22F	11356.79	-5918.29	29478.62	13968.83	-4707.23	31272.73
23A	19583.86	2091.42	36390.54	16430.84	-1658.94	34425.99
3	4204.09	-10402.1	18479.71	7894.13	-7770.29	22603.41
31	9875.27	-11992.4	31522.79	3307.89	-21515.5	26206.01
6A	-4723.36	-25570.3	16771.78	-1870.31	-24190.9	19805.18
6D	-7461.26	-28554.4	14396.92	-15199.3	-37823.2	7751.2
7F	24685.63	3471.88	46572.29	23255.71	1374.87	44902.99
8	9737.81	-3884.88	23393.2	13956.39	-1263.67	28318.91
9N	14296.32	-2889.44	31184.56	12904.55	-5848.54	30327.91
Undefined***	2907.8	-9633.08	15320.17	1982.93	-12155.3	14910.33

*Adjusted coefficient: adjusted for human immunodeficiency virus (HIV) status and age group
**HDI: Highest Density Interval of posterior distributions with "-L" = lower bound and "-H" = upper bound
***Undefined serotypes include pool G serotypes (29, 34, 35, 42 and 47), non-typables and serotypes not distinguishable by polymerase chain reaction

S6 Table Associations of serotypes with TNF-α concentrations

	Univariate			Adjusted*		
Serotype	**coefficient**	**HDI-L****	**HDI-H****	**coefficient**	**HDI-L****	**HDI-H****
10A	*Reference*	*Reference*	*Reference*	*Reference*	*Reference*	*Reference*
12F	1.42	-1.21	4.04	2.34	-0.77	5.17
14	1.59	-1.68	4.76	2.22	-1.22	5.58
15B/C	2.16	-0.68	5.11	2.71	-0.56	5.76
16F	0.55	-3.36	4.67	1.12	-3.09	5.31
18A	5.73	1.65	9.88	6.22	2.08	10.31
19A	1.2	-2.08	4.4	1.02	-2.36	4.46
19F	-0.9	-4.18	2.29	0.07	-3.69	3.53
22A	0.18	-3.79	4.28	1.46	-3.28	6.28
22F	0.24	-2.91	3.65	0.77	-2.61	4.17
23A	-0.86	-4.11	2.38	-0.99	-4.41	2.53
3	0.31	-2.32	2.99	1.02	-2.02	4.08
31	2.13	-1.9	6.05	3.4	-1.3	8.18
6A	3.48	-0.46	7.41	4.02	-0.2	8.07
6D	-2.48	-6.42	1.57	-3.21	-7.5	1.21
7F	0.67	-3.46	4.57	0.31	-4.01	4.37
8	1.37	-1.28	3.88	2.14	-0.61	4.96
9N	-0.09	-3.3	3.1	-0.12	-3.62	3.57
Undefined***	-0.02	-2.38	2.45	0.18	-2.42	2.82

*Adjusted coefficient: adjusted for human immunodeficiency virus (HIV) status and age group
**HDI: Highest Density Interval of posterior distributions with "-L" = lower bound and "-H" = upper bound
***Undefined serotypes include pool G serotypes (29, 34, 35, 42 and 47), non-typables and serotypes not distinguishable by polymerase chain reaction

S7 Table Associations of serotypes with case fatality

	Univariate			Adjusted*		
Serotype	**coefficient**	**HDI-L****	**HDI-H****	**coefficient**	**HDI-L****	**HDI-H****
10A	*Reference*	*Reference*	*Reference*	*Reference*	*Reference*	*Reference*
12F	-0.33	-2.68	1.86	-0.49	-2.91	1.73
14	-1.97	-6.09	0.64	-1.4	-5.92	1.76
15B/C	2.94	0.34	7.35	2.52	-0.09	6.69
18A	-1.44	-5.79	1.52	-1.83	-6.05	1.17
19A	1.91	-1.18	6.3	1.69	-1.53	6.12
19F	-1.51	-5.89	1.5	-1.83	-6.18	1.28
22F	1.9	-1.22	6.19	1.57	-1.32	5.79
23A	0.22	-2.11	2.72	-0.6	-3.32	1.98
3	0.74	-1.11	2.61	0.41	-1.48	2.49
6A	1.84	-1.1	6.04	1.53	-1.47	5.94
7F	-1.47	-5.95	1.53	-0.68	-5	2.98
8	-0.89	-2.66	0.8	-0.65	-2.73	1.24
9N	-1.51	-5.72	1.57	-0.7	-5.13	2.91
Undefined***	-0.67	-2.28	0.84	-1.44	-3.17	0.28

*Adjusted coefficient: adjusted for human immunodeficiency virus (HIV) status and age group
**HDI: Highest Density Interval of posterior distributions with "-L" = lower bound and "-H" = upper bound
***Undefined: include pool G serotypes (29, 34, 35, 42 and 47), non-typables and serotypes not distinguishable by polymerase chain reaction
